# Clinical Epidemiology of Systolic and Diastolic Orthostatic Hypotension in Patients on Peritoneal Dialysis

**DOI:** 10.3390/jcm10143075

**Published:** 2021-07-12

**Authors:** Claudia Torino, Rocco Tripepi, Maria Carmela Versace, Antonio Vilasi, Giovanni Tripepi, Vincenzo Panuccio

**Affiliations:** 1 National Research Council—Institute of Clinical Physiology, Via Vallone Petrara snc, 89124 Reggio Calabria, Italy; ctorino@ifc.cnr.it (C.T.); rtripepi@ifc.cnr.it (R.T.); mcversace@ifc.cnr.it (M.C.V.); avilasi@ifc.cnr.it (A.V.); gtripepi@ifc.cnr.it (G.T.); 2 Nephology, Dialysis and Transplantation Unit—GOM “Bianchi-Melacrino-Morelli”, Via Vallone Petrara snc, 89124 Reggio Calabria, Italy

**Keywords:** orthostatic hypotension, peritoneal dialysis, ESKD, clinical outcomes

## Abstract

Blood pressure changes upon standing reflect a hemodynamic response, which depends on the baroreflex system and euvolemia. Dysautonomia and fluctuations in blood volume are hallmarks in kidney failure requiring replacement therapy. Orthostatic hypotension has been associated with mortality in hemodialysis patients, but neither this relationship nor the impact of changes in blood pressure has been tested in patients on peritoneal dialysis. We investigated both these relationships in a cohort of 137 PD patients. The response to orthostasis was assessed according to a standardized protocol. Twenty-five patients (18%) had systolic orthostatic hypotension, and 17 patients (12%) had diastolic hypotension. The magnitude of systolic and diastolic BP changes was inversely related to the value of the corresponding supine BP component (*r* = −0.16, *p* = 0.056 (systolic) and *r* = −0.25, *p* = 0.003 (diastolic), respectively). Orthostatic changes in diastolic, but not in systolic, BP were linearly related to the death risk (HR (1 mmHg reduction): 1.04, 95% CI 1.01–1.07, *p* = 0.006), and this was also true for CV death (HR: 1.08, 95% CI 1.03–1.12, *p* = 0.001). The strength of this association was not affected by further data adjustment (*p* ≤ 0.05). These findings suggest that independent of the formal diagnosis of orthostatic hypotension, even minor orthostatic reductions in diastolic BP bear an excess death risk in this population.

## 1. Introduction

Blood pressure (BP) modulation is a complex mechanism which involves the cardiovascular, nervous, renal, and endocrine systems [1]. While peripheral regulation allows the fine tuning of BP thanks to the contribution of mediators such as nitric oxide, endothelins, and tissue plasminogen activator, at the central level BP is regulated by changes in cardiac output and vascular tone, mediated by the sympathetic and parasympathetic components of the autonomic nervous system [2]. At the central level, the renal control of extracellular volume, pressure natriuresis, kallikrein–kinin, and renin–angiotensin–aldosterone systems allow long-term BP control, while short-term variations are mainly due to baroreceptor and chemoreceptor reflexes [2,3,4,5,6,7,8,9].

The shift from the supine to the upright positions translates into a rapid decrease in central blood volume, causing, through a cascade of events, a fall in BP [10]. This fall is counteracted by the rapid activation of the sympathetic nervous system via mechanoreceptors and chemoreceptors [3].

Dysautonomia [11,12] and fluctuations in blood volume [13] are hallmarks in patients with kidney failure requiring replacement therapy, an elderly, high-risk population. The higher mortality rate in such patients is partially explained by traditional risk factors and a higher rate of cardiovascular events; other factors include inflammation, alteration in mineral metabolism, volume expansion with the exacerbation of arterial hypertension/hypotension, and alteration of the sympathetic nervous system [14,15,16]. As defects in the sympathetic nervous system translate into orthostatic hypotension (OH) [17], considering that they are pervasive in kidney failure requiring replacement therapy patients [11,12], it is clear how OH is frequent in these patients.

OH has been associated with syncope [18], cardiovascular events [19,20,21,22,23,24,25], and mortality in the general population [26,27]. The same association with mortality was found in chronic hemodialysis (HD) treatment [28]. However, neither this relationship nor the impact of changes in blood pressure has been tested in patients on peritoneal dialysis (PD), a population with a peculiar hemodynamic and risk factor profile.

The aim of this retrospective study is to investigate the relationship between OH and orthostatic blood pressure changes with all-cause and cardiovascular (CV) mortality in a cohort of PD patients.

## 2. Materials and Methods

### 2.1. Study Population

**Figure 1:**
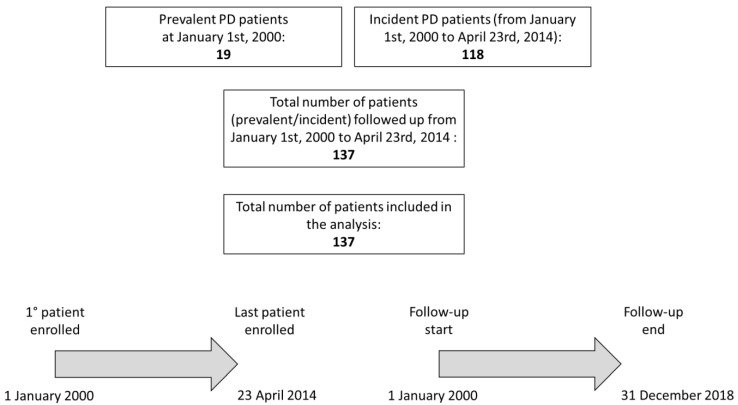
Flow of patients in the study.

The study population represents the prevalent and incident PD patients followed at our center from 1 January 2000 to 23 April 2014 (*n* = 137). Data included in this study were retrospectively collected using clinical records, according to the current ethical rules. In order to avoid selection bias, all patients on regular PD, either on 4 standard exchanges per day or on continuous cycling peritoneal dialysis, with response to orthostasis assessed and available at clinical chart review, were included. The study flow is described in Figure 1.

### 2.2. Measurement of the Response to Orthostasis

The response to orthostasis was evaluated at our center according to a standardized protocol of the European Society of Cardiology/European Society of Hypertension [29]. Briefly, systolic and diastolic BP (SDB and DBP, respectively) were measured three times after 10 min in a supine position and once after 1–2 min in an upright position. As no specific indications are recommended in the guidelines of ESC/ESH 2018 [29], according to the rules followed at our center, we used the last value for both supine SBP/DBP. OH was defined as a drop of ≥20 mmHg in SBP and/or ≥10 mmHg in DBP [29].

### 2.3. Laboratory Measurements

Blood sampling was performed at the day of assessment after an overnight fast. Cholesterol, albumin, calcium, phosphate, alkaline phosphatase, PTH (intact molecule), C-Reactive Protein (CRP), fibrinogen, and hemoglobin measurements were performed using standard methods in the routine clinical laboratory.

### 2.4. Study End-Points

In this paper, the association between OH and orthostatic SBP and DBP changes with overall and cardiovascular (CV) mortality was tested. Patients were followed-up from baseline (day of the measurement of the response to orthostasis) until death or censoring. Patients who underwent kidney transplantation or shifted from PD to hemodialysis (HD) were censored. Cardiovascular events were centrally adjudicated and classified as follows: stroke (ischemic or hemorrhagic) documented by computed tomography, magnetic resonance imaging, and/or clinical and neurological evaluation; transient ischemic attacks (TIA); myocardial infarction confirmed by serial changes in ECG and cardiac biomarkers; ECG-documented angina episodes; ECG-documented arrhythmia; unexpected, sudden death highly suspected as of cardiac origin. De novo chronic heart failure (CHF) was defined as CHF in a patient without CHF at baseline. To be classified as having CHF, patients had to show mild or more severe dyspnea during ordinary activities (NYHA class II or higher), plus evidence of anatomical/functional LV disease on echocardiography. Each cause of death was assessed by 3 independent physicians. In doubtful cases, diagnosis was attributed by consensus. During the review process, the investigator used all available medical information, including hospitalization forms and medical records. In the case of death occurring at home, family members and/or general practitioners were contacted to retrieve the cause of death.

### 2.5. Statistical Analysis

Considering the retrospective design of our study, no sample size calculation was made. However, in order to assess if data collection would be adequate to perform the planned survival analyses, we carefully checked the database before the statistical assessment. Among 137 patients, 69 manifested the event of interest (all-cause death) during the follow-up, allowing us to build survival models including up to 7 variables. This made our cohort satisfactory for our analysis.

Data were expressed as mean ± standard deviation (normally distributed data), median and inter-quartile range (non-normally distributed data), or as percent frequency (categorical data). Comparisons among groups were made by Student’s *t*-Test, Mann–Whitney U, or Chi-square test, as appropriate. Pearson’s correlation analyses were performed to investigate the correlates of magnitude of SBP and DBP changes. Survival analyses were performed to investigate the association between OH, systolic and diastolic changes, and the considered outcomes. Bivariate Cox regression models included SBP and DBP changes, together with the corresponding supine measurement (SBP or DBP). In multivariate models, age, gender, diabetes, and cardiovascular comorbidities were also included. The association between antihypertensive drugs and changes in blood pressure was tested, building a multiple linear regression model adjusted for all antihypertensive drugs classes (calcium channel blockers, ACE inhibitors, sartans, alpha or beta blockers, clonidine, furosemide). To overcome the issue of model overfitting when CV death was included in survival models as an outcome variable, we repeated multivariate analyses using the van Houwelingen and le Cessie heuristic shrinkage estimate [30]. As the number of missing values was less than 5% for each variable, in multiple models we replaced missing data with the mean or median values (according to the data distribution). The potential effect modification by diabetes, markers of mineral metabolism (calcium, phosphate, PTH), dialysis adequacy (KT/V), or inflammation (CRP) on the relationship between OH and death/CV death was investigated by standard analyses through creating appropriate multiplicative terms in Cox regression analyses. Statistical analysis was performed using standard statistical packages (SPSS for Windows, Version 24, Chicago, IL, USA).

## 3. Results

The main demographic, somatometric, clinical, and biochemical characteristics of the study population are detailed in Table 1. Thirty-four patients presented with OH upon clinical chart assessment.

**Figure 2:**
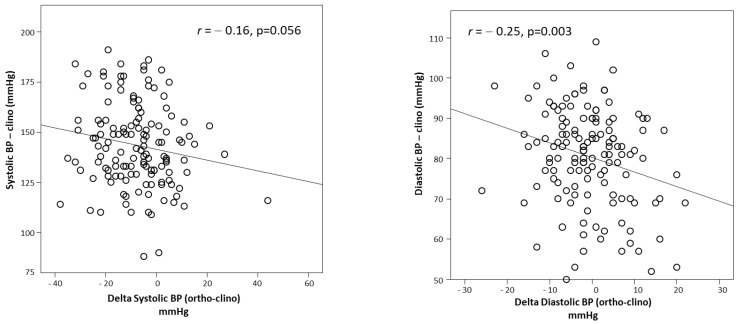
Correlation between changes in systolic (**left panel**) and diastolic (**right panel**) blood pressure (BP) from supine to upright positions and the corresponding supine BP component.

Orthostatic BP excluded, no differences were found between patients with or without OH, except for KT/V, which tended to be lower in the first group. Average supine BP was 144 ± 21/81 ± 12 mmHg, and upright BP was 136 ± 23/80 ± 13 mmHg. The average postural change was −8 ± 13 (systolic)/−1 ± 8 mmHg (diastolic). Twenty-five patients (18%) had systolic, and 17 patients (12%) had diastolic OH. The magnitude of SBP and DBP changes were inversely related to the value of the corresponding supine BP component (*r =* −0.16, *p* = 0.056 (systolic) and *r =* −0.25, *p* = 0.003 (diastolic), respectively) (Figure 2). One hundred and twenty-five patients (91.2%) were treated with various antihypertensive drugs (calcium channel blockers, ACE inhibitors, sartans, alpha or beta blockers, clonidine, furosemide).

### Survival Analyses

During a median follow-up of 37 months (interquartile range: 22–62 months), 69 patients died (19/34 (56%) with OH and 50/103 (49%) without OH). The corresponding Incidence Rates (IR) were 16.99/100 PY and 12.55/100 PY, with an IRR of 1.45 (*p* = 0.26). Among them, 45% died due to CV causes. The IR of CV death was 8.94 in patients with OH, and 5.27 in patients without OH (IRR: 1.70, *p* = 0.16). Forty-four patients (9 with OH and 35 without OH) shifted to HD, and 17 underwent kidney transplantation (4 with OH and 13 without OH). Univariate Cox regression models showed no association between OH and all-cause/CV mortality (HR_all-cause_: 1.48, 95% CI: 0.86–2.53, *p* = 0.15; HR_CV_: 1.74; 95% CI: 0.81–3.72, *p* = 0.16) (Figure S1).

However, in Bivariate Cox regression models, orthostatic changes in DBP, adjusted for supine DBP, were linearly related to the death risk (HR: 1.04, 95% CI 1.01–1.07, *p* = 0.006), and this was also true for CV death (HR: 1.08, 95% CI 1.03–1.12, *p* = 0.001). Further data adjustment for other potential confounders (age, gender, diabetes, and background CV comorbidities) did not materially affect the strength of the association between diastolic BP changes and the risk of all-cause (HR: 1.03, 95% CI 1.00–1.07, *p* = 0.05) and CV death (HR: 1.09, 95% CI 1.03–1.14, *p* = 0.002). Considering the low number of CV death, in order to overcome the issue of model overfitting we repeated multivariate analyses using the shrinkage approach. After shrinkage, the results remained roughly the same (Table 2). Conversely, orthostatic changes in SBP were unrelated to mortality (Table 2). In order to investigate the effect of antihypertensive drugs on changes in blood pressure, a multiple linear regression model (dependent variable: changes in SBP), adjusted for all antihypertensive drugs classes (calcium channel blockers, ACE inhibitors, sartans, alpha or beta blockers, clonidine, furosemide), was built. In the multivariate linear regression analysis, only the use of B-blockers resulted in being directly related to DBP changes (β = 0.277, *p* = 0.001). On the basis of this analysis, we introduced beta blockers into the multiple Cox models of all-cause and CV mortality, and the links between DBP changes and these two endpoints remained substantially unchanged (all-cause mortality: HR 1.04, 95% CI: 1.00–1.07, *p* = 0.05; CV mortality: HR 1.01, 95% CI: 1.04–1.17, *p* = 0.001). Of note, in these survival models, the use of B-blockers failed to be related (all-cause mortality: HR 0.87, 95% CI: 0.42–1.77, *p* = 0.69; CV mortality: HR 0.45, 95% CI: 0.14–1.43, *p* = 0.18).

**Figure 3:**
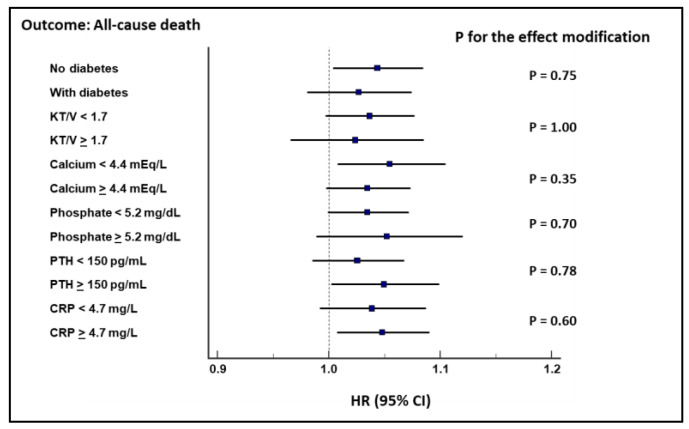
Cox regression analysis showing no effect modification of the considered variables on the association between diastolic changes and all-cause death.

**Figure 4:**
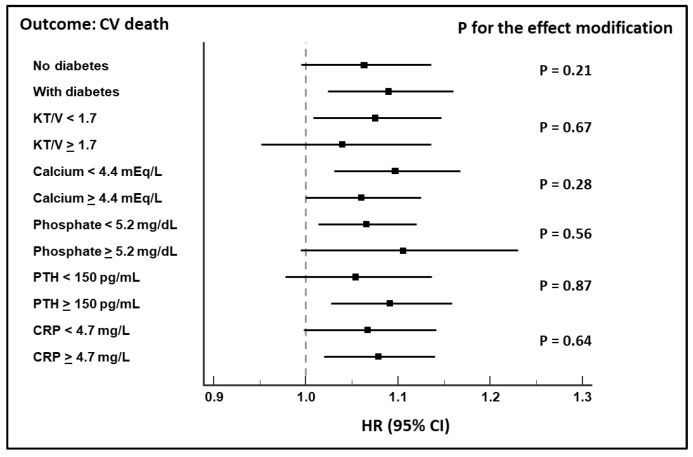
Cox regression analysis showing no effect modification of the considered variables on the association between diastolic changes and CV death.

No effect modification by diabetes, alteration of markers of mineral metabolism, dialysis adequacy, or inflammation was found (Figure 3 and Figure 4).

## 4. Discussion

In this study, we investigated the impact of OH and systolic and diastolic orthostatic changes on clinical outcomes in patients on peritoneal dialysis. Our results show for the first time a linear association between low orthostatic changes in diastolic blood pressure and both all-cause and cardiovascular mortality in this cohort.

Patients affected by renal failure are often affected by autonomic neuropathy [11,12,31,32], with autonomic failure being the main cause of postural and generalized hypotension [33]. The prevalence of OH ranges from 5% to 50%, according to age, population, and threshold used [34,35,36]. In our cohort, 25 out of 137 patients (18%) had systolic OH, and 17 out of 137 (12%) had diastolic hypotension. These results are in line with a study published by Bhat et al., reporting a prevalence of OH of 12.6% in stable CKD patients [37].

The association between impaired orthostatic BP stabilization and adverse outcomes, such as fall and syncope, has been well documented in different populations, including patients with renal impairment [38,39,40]. Furthermore, OH, in turn, increases the risk of chronic kidney disease in a middle-aged population [41]. OH increases the risk of all-cause mortality both in the general population [26,27] and in patients affected by CKD [28,42]. Less documented is the link between OH and cardiovascular events, even though data available in literature suggest a positive association with heart failure, myocardial infarction, and stroke in the general population [25,43,44]. A positive association has also been found with aortic stiffness, while the relationship with central systolic pressures has not been completely clarified [45,46].

To our knowledge, little is known about the effect of OH or changes in blood pressure on clinical outcomes, including mortality, in PD patients, a frail population with a peculiar hemodynamic profile.

In our study, we focused on the magnitude of systolic and diastolic changes from the supine to upright position, finding an inverse correlation between these values and the corresponding supine BP component.

We found no association between OH and all-cause/CV death. However, both at univariate and multivariate analysis, orthostatic changes in DBP, but not in SBP, were linearly related to all-cause and CV death, with a risk excess of 4% and 8%, respectively, for each increase of 1 mmHg in the difference between supine and upright diastolic BP. In other words, a higher BP fall between supine and upright position was associated with worse outcomes. This relationship did not change when therapies with Beta blockers, the only antihy-pertensive drugs associated with changes in SBP, were included in the model.

No effect modification by diabetes was found in the link between orthostatic DBP changes and the considered outcomes, suggesting dysfunction of the autonomic nervous system, and not diabetic neuropathy, as a cause of OH. Similarly, this association was not influenced by the alteration of the mineral metabolism, dialysis adequacy, or inflammation.

PD patients, as well as other patients affected by chronic disease, are elderly, and older age is a well-known risk factor for OH. Furthermore, the alteration of cardiac geometry, such as left ventricular hypertrophy, is more severe in long-term peritoneal than in HD patients. Similarly, the left atrial natriuretic factor (ANF), the atrial volume, and the number of antihypertensive drugs are significantly higher in PD patients [47]. A peculiarity of PD patients is the increase in intra-abdominal pressure after abdominal filling with dialysis fluid, with significant changes in some hemodynamic parameters, such as inferior vena cava pressure, while the post-dialysis changes regard central parameters such as cardiac index and pulmonary artery pressure [48]. These characteristics, together with hormonal and neuromediator alterations, could explain the OH in this population of patients. Most patients with OH are asymptomatic or have few nonspecific symptoms, thus accounting for the high rate of unrecognized cases [49].

Our study has limitations—first of all, the small sample size. However, considering the length of the follow-up and the fact that half of the patients experienced the event of interest, our cohort can be considered satisfactory. Secondly, the patients enrolled were followed up in a single dialysis center, thus our results cannot be generalized. Third, despite more than half of our patients experiencing the outcome of interest during the follow-up, the number of the events collected was relatively small, allowing us to adjust only for a limited set of confounders. Furthermore, smoking habit was available only in a limited number of subjects. In addition, OH was diagnosed using only baseline BP measurements, as longitudinal data were not available. However, BP measurements were evaluated according to a standardized protocol of ESC/ESH [29] and are in line with the latest KDIGO guidelines [50], which, even though not focused on the diagnosis of OH, recommends that the patient relax for at least 5 min before BP measurement. Finally, although OH is associated with a higher risk of falls [26], during the follow-up no falls leading to significant clinical consequences (fractures, trauma, hospital admissions) were recorded in clinical records. However, we cannot exclude that falls leading to minor consequences, and thus not recorded, occurred during the follow-up.

In spite of these limitations, our study is the first showing an association between orthostatic BP fall and mortality in PD patients. As OH is a more robust predictor of cardiovascular events than night-time reverse dipping in the elderly [51], and, unlike dipping, OH does not require Ambulatory Blood Pressure Measurements to be detected, our results highlight the appropriateness of introducing the measurement of blood pressure falls in clinical practice, as it could easily allow the identification of patients at high risk and the start of the appropriate therapy.

Therapeutic strategies range from patients’ educational program to drugs therapy. For example, patient training focused on recognizing OH and avoiding any conditions that can aggravate it (i.e., suddenly assuming a standing position or dehydration during illnesses, such as fever or gastroenteritis) is extremely helpful. Another approach consists of educating patients to prevent BP decreases using countermeasures, such as muscle tension and squatting [52]. In selected cases, some drugs such as droxidopa and midodrine could help the management of OH. Finally, the use of short acting antihypertensive drugs and night-time administration can help prevent hypotensive episodes.

## 5. Conclusions

In conclusion, orthostatic SBP and DBP changes (but not OH) are associated with adverse outcomes in PD patients. Further studies, specifically designed for the purpose, are needed to investigate this phenomenon.

## Figures and Tables

**Table 1:** Main demographic, somatometric, and clinical characteristics in patients as divided according to OH.

	With OH (*n* = 34)	Without OH (*n* = 103)	*p* Value
Age (years)	66 ± 12	65 ± 14	0.57
BMI (kg/m^2^)	27 ± 4	27 ± 4	0.48
Male sex N (%)	25 (74)	62 (60)	0.16
Diabetics N (%)	15 (44)	39 (38)	0.29
With cardiovascular comorbidities	17 (50)	44 (43)	0.46
KT/V	1.7 ± 0.3	1.8 ± 0.4	0.06
On antihypertensive treatment N (%)	32 (94)	93 (90)	0.49
Systolic BP clino (mmHg)	147 ± 20	143 ± 21	0.27
Diastolic BP clino (mmHg)	82 ± 12	80 ± 12	0.45
Systolic BP ortho (mmHg)	126 ± 23	139 ± 21	**0.004**
Diastolic BP ortho (mmHg)	74 ± 13	82 ± 12	**0.002**
Cholesterol (mg/dL)	176 ± 46	182 ± 45	0.49
Hemoglobin (g/dL)	11.4 ± 2.0	11.5 ± 1.4	0.65
Albumin (g/dL)	3.6 ± 0.6	3.6 ± 0.4	0.99
CRP (mg/L)	4.3 (3.1–16.3)	5.0 (3.3–12.1)	0.60
Calcium (mEq/L)	4.4 ± 0.6	4.4 ± 0.5	0.96
Phosphate (mmol/L)	5.3 ± 1.3	5.4 ± 1.8	0.79
PTHi (pg/mL)	210 (47–321)	146 (57–247)	0.85

Data are expressed as mean ± SD, median, and inter-quartile range or as percent frequency, as appropriate. Bold: significant *p*-Values

**Table 2:** Survival analysis showing the association between changes in DBP and all-cause and CV death.

	All-Cause Death	CV Death	CV Death (after Shrinkage)
Changes in SBP (1 mmHg) *	1.01 (0.99–1.03), *p* = 0.35	1.01 (0.99–1.03), *p* = 0.22	---
Changes in SBP (1 mmHg) **	1.01 (0.99–1.02), *p* = 0.59	1.02 (0.99–1.04), *p* = 0.26	1.01 (0.99–1.04), *p* = 0.37
Changes in DBP (1 mmHg) *	1.04 (1.01–1.07), *p* = 0.006	1.08 (1.03–1.12), *p* = 0.001	---
Changes in DBP (1 mmHg) **	1.03 (1.00–1.07), *p* = 0.05	1.09 (1.03–1.14), *p* = 0.002	1.07 (1.02–1.13), *p* = 0.01

* adjusted for clino SBP or DBP; ** adjusted for clino SBP or DBP, age, gender, CV comorbidities, diabetes.

## Data Availability

The data presented in this study are available on request from the corresponding author. The data are not publicly available as collected by using clinical records.

## Supplementary Materials

The following are available online at https://www.mdpi.com/article/10.3390/jcm10143075/s1, Figure S1: Kaplan-Meier curves showing no association between OH and all-cause (left panel) and CV death (right panel).
