# Supplementary material for: Clinical Epidemiology of Systolic and Diastolic Orthostatic Hypotension in Patients on Peritoneal Dialysis
Source: J Clin Med. 2021 Jul 12;10(14):3075. doi: 10.3390/jcm10143075 (PMC8304693; doi:10.3390/jcm10143075)
Supplement: Supplementary file 1 [file jcm-10-03075-s001.zip › Suppl.Fig.1.pptx]

## Slide 1
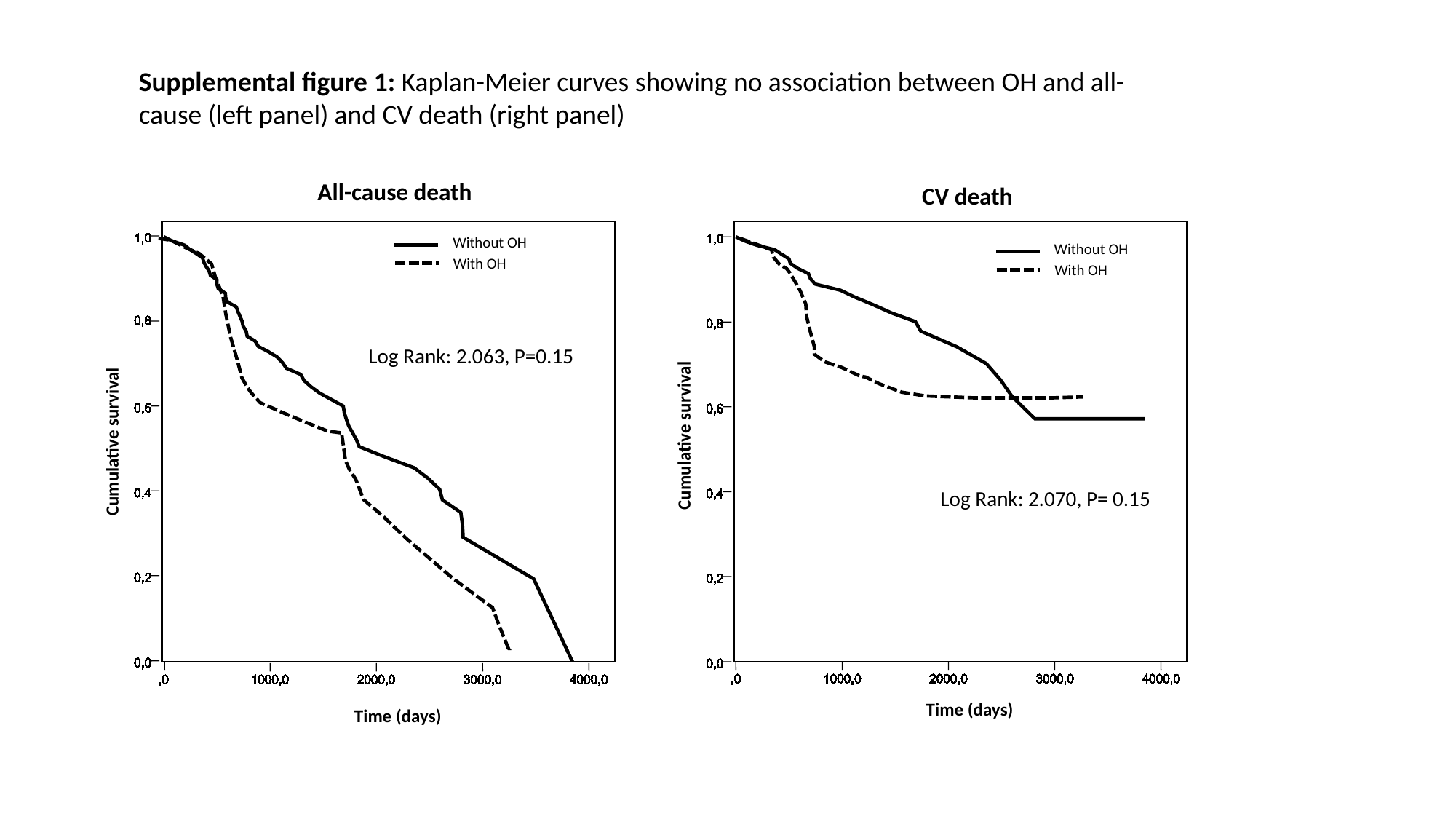

Supplemental figure 1: Kaplan-Meier curves showing no association between OH and all-cause (left panel) and CV death (right panel)
All-cause death
CV death
Without OH
Without OH
With OH
With OH
Log Rank: 2.063, P=0.15
Cumulative survival
Cumulative survival
Log Rank: 2.070, P= 0.15
Time (days)
Time (days)
